# Assessment of the release of metals from cigarette butts into the environment

**DOI:** 10.1371/journal.pone.0260111

**Published:** 2021-11-18

**Authors:** Carla Roselli, Ivan Fagiolino, Donatella Desideri, Davide Sisti, Maria Assunta Meli

**Affiliations:** 1 Department of Biomolecular Sciences, Università di Urbino Carlo Bo, Urbino (PU), Italy; 2 GRUPPO C.S.A. S.p.A., Rimini (RN), Italy; Indian Institute of Technology Patna, INDIA

## Abstract

Cigarette butts are known to contain toxic metals which pose a potential threat to the environment and human health. The seriousness of this threat is largely determined by the leachability of these toxic metals when the butts are exposed to aqueous solutions in the environment. The aims of this study were to determine the presence and mobility of toxic and non-toxic elements found in discarded cigarette butts; to relate this mobility to two different contact situations with leaching liquids: tumbling and trampling (batch test) and percolation in a static position (column test); and finally, to verify possible variations in solubility by simulating different environmental systems. Five leachants with different pH values were used to simulate various environmental conditions The concentrations of the solubilized metals were determined by inductively coupled plasma-atomic emission spectrometry (ICP-AES) and inductively coupled plasma-mass spectrometry (ICP-MS). CH_3_COOH pH 2.5 showed the greatest capacity to dissolve many elements. On the contrary, weakly acidic or alkaline environments did not favor the leachability of the elements. The best extraction capacity of the column with respect to the batch is statistically significant (p <0.05) for the elements Al, Fe, Ni and Zn, while the batch for P, Si, S. Pb, Cd, As were not detectable in cigarette butts, while Hg had an average concentration of 0.0502 **μ**g/g. However, Hg was < LOD in all different leachants.

## Introduction

Tobacco products are classified among the most dangerous carcinogens [1]. Health risks associated with smoking are related to the presence of several carcinogens and toxicants in cigarettes, including polynuclear aromatic hydrocarbons, N-nitrosamine, natural radionuclides and toxic metals and metalloids [2, 3]. These dangerous elements are found in the tobacco plants themselves, in the chemicals formed during the curing, fermenting, processing, and aging of tobacco as well as in cigarettes and their discarded butts [3]. The accumulation of such metals in tobacco plants varies according to soil metal content, pH and others factors. Mineral phosphate and nitrate fertilizers are also known as potential sources of heavy metals in soil. In addition, tobacco plants acquire metals from airborne contaminants and polluted water [4]. Some metals pass readily into the bloodstream during smoking and many accumulate in specific organs [5].

Cigarette butts, among the most common forms of litter, are a potential source of toxic substance contamination in the environment [6]. Specifically, these discarded butts contain arsenic and heavy metals, nicotine and polycyclic aromatic hydrocarbons [2, 7]. Although a single cigarette stub does not pose a significant threat to the environment, the cumulative effect of large quantities of butts discarded in a particular area may indeed pose a threat to local organisms when their harmful contents leach into the environment [8–11, 12]. While it is true that the metal tolerance of some species (eg, bioaccumulators) can be enhanced by trace and heavy metal contamination in soil and water, other organisms can be adversely affected by such contamination [13–15]. Moreover, biological responses to contamination can be altered by environmental conditions, such as pH, which affect the mobility of metals in soils and the bioavailability of those metals to plants [16, 17]. Hence, it is important to determine the elemental composition of different types of cigarette butts and to investigate their leaching behaviour in order to properly assess their toxicity and to protect the environment and humans against the potential threat that they pose.

Although tobacco plants readily accumulate many toxic metals from soils (in particular, cadmium), toxic metals have not been investigated as thoroughly as nicotine and tobacco-specific nitrosamine [3, 12]. In addition, few investigations have sought to evaluate the leachability of metals found in cigarette butts that are discarded in the environment [18]. Leachability tests measure the potential availability of components from solid, mainly inorganic elements, and allow us to draw a distinction between extractable substances and those that will be retained in the matrix under natural outdoor conditions [19]. Leaching tests therefore provide more than just information on component composition, and they are indispensable to make an accurate assessment of the true threat posed by any contaminant to the environment and human health [20]. Comparisons have been drawn between leaching tests designed for different wastes, [16, 19, 21, 22] but there is a lack of information on the mobility to the environment of the elements in discarded cigarette butts.

## Materials and methods

### Samples and sampling

Discarded cigarette butts of eight popular cigarette brands in Italy were analyzed. The cigarette stubs were collected from covered collectors that had been placed near the University of Urbino (Marche region, Central Italy). The sampling was not performed after local precipitation events in order to limit the loss of components in the butts before sampling. After collection, the filters were manually separated from any remaining tobacco and then stored in disposable plastic containers.

### Sample preparation for the elemental analysis

The sample for elemental analysis (about 3.3 g) was constituted by 16 butts (2 butts for each brand). The sample was finely shredded and homogenized; its dissolution was carried out according to the EPA 3052 1996 method proposed by the Environmental Protection Agency [23]. Five hundred milligrams (dry weight) of the sample was digested in a mixture of 7 mL concentrated nitric acid, 3 mL 30% hydrogen peroxide and 0.2 mL 40% hydrofluoric acid for 15 minutes using microwave heating with a suitable laboratory microwave system. After cooling, 1 mL 6% boric acid was added. The digestate was filtered through 0.45-**μ**m pore size filter paper; after washing, the solution was brought up to 50 mL. All of the chemicals used in the sample treatment were suprapure grade (Fluka, for trace analyses, Merck, suprapure, Aldrich, for trace analyses); ultrapure water was used for all solutions.

### Leaching procedure

Leaching occurs when there is contact between a solid sample and a liquid (leachant). Many physical and chemical factors such as leachant composition, pH, leaching procedure, complexing agents, oxidants and reducing agents, time of contact and liquid-solid ratio can affect the element behaviour [16]. Within the framework of European leaching tests [24], well-defined standard leaching test methods have been developed to obtain information on the short and long-term leaching behaviour of waste materials [25]. The two-stage batch test according to EN 12457–3 [26], and the up-flow percolation test according to prEN14405 [25] are examples of such methods. Demineralized water was used as a leachant in these tests so as not to trigger processes such as oxidation and acidification [27].

In the present work, the leachates of cigarette butts were analyzed according to modified versions of two different procedures found in the literature [17, 24, 28]: a) batch extraction and b) column percolation. These methods were adjusted for the small dimensions of the tested samples. Instead of demineralized water, five leaching solutions were used to simulate a closed system environment: A) 0.4M MgCl_2,_ pH = 6.5; B) CH_3_COOH and CH_3_COONa pH 5; C) CH_3_COOH, pH 2.5; D) 0.02M HNO_3_ and 30% H_2_O_2_ (ratio 3:5), pH <1; E) NaOH pH 8. We used a diluted magnesium chloride solution to test fine grained soil; acidified reagent water to simulate acid rainwater; acetic acid, since organic acids are to be found in soil; nitric acid and hydrogen peroxide to simulate extreme acid and oxidant conditions; and alkaline solution to simulate a leachate coming from a landfill. All these leachants were prepared with reagents of analytical grade (BDH, Aldrich and Merck). The pH of these solutions was determined within 0.05 of the desired unit with a pHmeter Crison 524 (Crison Instruments, SA Riera Principal, 34, 36, E-08328 Alella, Spain).

In the batch extraction, 11 ml of leachant was added to 3.3 g of the sample (whole cigarette butts) in a plastic bottle; after mixing in a rotator, the two phases (solid sample and extraction solution) were separated by filtration (cellulose nitrate membrane filters 0.45 **μ**m) and saved. Subsequently the solid residue was again added to 11 ml of the same fresh leachant in the original bottle. After leaching, separation and filtration, a third step was performed under the same conditions. Fifteen solutions (three fractions for each of five leachants) were obtained. The ratio between the liquid phase (L) and solid phase (S) was 3.3 ml/g for every step (11 ml for 3.3g of the sample) and 10 ml/g (33 ml for 3.3 g of the sample) for all three steps.

In the column extraction, 33 ml of leachant was fluxed through a glass column containing 3.3 g of the sample (whole cigarette butts) (accumulated ratio L/S = 10 ml/g); the leachate was collected in three fractions (11 ml per fraction). Fifteen solutions (three fractions for each of five leachants) were obtained. The procedures of batch extraction and column percolation (performed in double) are described in detail in Desideri et al. [18].

### Elemental analysis

In the solutions derived from butt dissolution and leachability tests, an elemental analysis was carried out by EPA 6010D 2014 [29] and EPA 6020B 2014 [30] for liquid matrix (Table 1).

**Table 1:** Method of element analysis for the solid and liquid matrices with the relative quantification limit (LOQ) and detection limit (LOD).

Element	Method for solid matrix	LOQ (mg/g)	LOD (mg/g)	Method for liquid matrix	LOQ (mg/ml)	LOD (mg/ml)
**Al**	EPA 3052 1996 + EPA 6010D 2014	0.1	0.03	EPA 6020B 2014	0.005	0.002
**Sb**	EPA 3052 1996 + EPA 6010D 2014	5	1.67	EPA 6020B 2014	0.001	0.0003
**Aa**	EPA 3052 1996 + EPA 6010D 2014	1	0.33	EPA 6020B 2014	0.001	0.0003
**Ba**	EPA 3052 1996 + EPA 6010D 2014	0.5	0.17	EPA 6020B 2014	0.0005	0.0002
**Cd**	EPA 3052 1996 + EPA 6010D 2014	0.5	0.17	EPA 6020B 2014	0.001	0.0003
**Ca**	EPA 3052 1996 + EPA 6010D 2014	0.5	0.17	EPA 6020B 2014	0.50	0.20
**Co**	EPA 3052 1996 + EPA 6010D 2014	0.5	0.17	EPA 6020B 2014	0.001	0.0003
**Cr**	EPA 3052 1996 + EPA 6010D 2014	1	0.33	EPA 6020B 2014	0.001	0.0003
**Fe**	EPA 3052 1996 + EPA 6010D 2014	5	1.67	EPA 6020B 2014	0.005	0.0017
**Ps**	EPA 3052 1996 + EPA 6010D 2014	0.5	0.17	EPA 200.7 2001	0.01	0.003
**Mg**	EPA 3052 1996 + EPA 6010D 2014	0.5	0.17	EPA 6020B 2014	0.50	0.20
**Mn**	EPA 3052 1996 + EPA 6010D 2014	0.5	0.17	EPA 6020B 2014	0.001	0.0003
**Ni**	EPA 3052 1996 + EPA 6010D 2014	0.5	0.17	EPA 6020B 2014	0.0005	0.00017
**Pb**	EPA 3052 1996 + EPA 6010D 2014	0.5	0.17	EPA 6020B 2014	0.001	0.0003
**K**	EPA 3052 1996 + EPA 6010D 2014	0.5	0.17	EPA 6020B 2014	0.50	0.20
**Cu**	EPA 3052 1996 + EPA 6010D 2014	1	0.33	EPA 6020B 2014	0.001	0.0003
**Si**	EPA 3052 1996 + EPA 6010D 2014	10	3.33	EPA 200.7 2001	0.01	0.003
**Sn**	EPA 3052 1996 + EPA 6010D 2014	0.2	0.07	EPA 6020B 2014	0.001	0.0003
**Sr**	EPA 3052 1996 + EPA 6010D 2014	0.5	0.17	EPA 6020B 2014	0.001	0.0003
**Ta**	EPA 3052 1996 + EPA 6010D 2014	0.1	0.033	EPA 6020B 2014	0.001	0.0003
**Te**	EPA 3052 1996 + EPA 6010D 2014	0.5	0.17	EPA 6020B 2014	0.001	0.0003
**Ti**	EPA 3052 1996 + EPA 6010D 2014	0.5	0.17	EPA 6020B 2014	0.001	0.0003
**Th**	EPA 3052 1996 + EPA 6020B 2014	0.1	0.033	EPA 6020B 2014	0.0025	0.0008
**Zn**	EPA 3052 1996 + EPA 6010D 2014	0.5	0.17	EPA 6020B 2014	0.005	0.0017
**S**	EPA 3052 1996 + EPA 6010D 2014	1	0.33	EPA 200.7 2001	0.1	0.03
**Ce**	EPA 3052 1996 + EPA 6020B 2014	1	0.33	EPA 6020B 2014	0.001	0.0003
**La**	EPA 3052 1996 + EPA 6020B 2014	1	0.33	EPA 6020B 2014	0.001	0.0003
**Rb**	EPA 3052 1996 + EPA 6020B 2014	1	0.33	EPA 6020B 2014	0.001	0.0003
**U**	EPA 3052 1996 + EPA 6020B 2014	1	0.33	EPA 6020B 2014	0.001	0.0003
**Hg**	EPA 7473 2007	0.0005	0.00017	EPA 6020B 2014	0.0001	0.00003

In EPA 6010D 2014, element determination was carried out by inductively coupled plasma-atomic emission spectrometry (ICP-AES), which may be used for a multi-elemental determination of trace elements in solutions. The quantification limits (LOQ) and detection limit (LOD) are shown in Table 1.

In EPA 6020B 2014, element determination was carried out by the measurement of ions produced by radio-frequency inductively coupled plasma (ICP-MS) using an X Series II ICP-MS (Thermo Fisher Scientific Inc, NYSE TMO) with an Octopole Reaction System. The LOQ and LOD are shown in Table 1. Details on instrumental operating conditions of ICP-AES and ICP-MS methods are provided in Meli et al. [31].

P, Si and S were also determined by EPA 200.7 2001 (ICP-AES) [32]. The LOQ and LOD of three elements are shown in Table 1. Hg in solid butts was determined by EPA 7473 2007 [33], using thermal decomposition, amalgamation and atomic absorption spectrometry; the LOQ value was 0.0005 **μ**g/g and LOD 0.00017 **μ**g/g.

### Quality control

A blank sample was also prepared in order to take into account the possible impurity of reagents and release from containers and equipment. Interference needs to be assessed and valid corrections applied or data flagged to indicate problems. The accuracy of the method was evaluated using recovery tests with a laboratory control system (LCS) consisting of a blank sample to which there are known quantities of analytes. The average analytical standard error obtained was 20% compared to the reported certified materials.

## Results

### Elemental composition of cigarette butts

For the sample (16 cigarette butts from eight common brands), Table 2 shows the element concentration obtained with five replicates, the mean concentration, the standard deviation (SD) and the relative standard deviation (RSD%) compared to toxic elements (Al, As, Ba, Cd, Ce, Hg, La, Ni, Pb, Rb, Sb, Sn, Sr, Te, Ti, Tl, Th, and U) and other non-toxic elements (Ca, Co, Cr tot, Cu, Fe, K, Mg, Mn, P, S, Si and Zn). Twenty-one of the 30 elements that were analyzed showed a concentration > LOQ being completely or partially retained in the cigarette butts.

**Table 2:** Element concentrations (mg/g) (5 replicates), mean, standard deviation (SD) and relative standard deviation (RSD%) in 16 cigarette butts from eight common brands.

	Element	1	2	3	4	5	Mean	SD	RSD%
**Non-essential**	Al	866	1007	699	877	1198	929	186	20
**or toxic**	Sb	< 5	< 5	< 5	< 5	< 5	<5	-	-
	As	< 1	< 1	< 1	< 1	< 1	< 1	-	-
	Ba	3.9	3.8	3.4	3.5	5.1	3.9	0.68	17
	Cd	< 0.5	< 0.5	< 0.5	< 0.5	< 0.5	< 0.5	-	-
	Ce	< 1	< 1	< 1	< 1	< 1	< 1	-	-
	La	< 1	< 1	< 1	< 1	< 1	< 1	-	-
	Pb	< 0.5	< 0.5	< 0.5	< 0.5	< 0.5	< 0.5	-	-
	Hg	0.0594	0.0530	0.0423	0.0491	0.0474	0.0502	0.0100	20
	Ni	0.60	0.70	< 0.50	0.60	0.90	0.70	0.14	20
	Rb	3.0	2.0	2.0	2.0	2.0	2.2	0.45	20
	Sr	7.8	7	7.9	8	8.3	7.80	0.48	6.5
	Te	23.7	21.5	19.6	20.2	23.4	21.7	1.84	8.5
	Ta	0.60	0.30	0.40	0.50	0.50	0.46	0.11	24
	Th	< 0.1	< 0.1	< 0.1	< 0.1	< 0.1	< 0.1	-	-
	Sn	1.6	1.6	1.3	1.1	0.90	1.3	0.31	24
	Ti	5330	5524	5638	5242	4602	5267	403.3	7.7
	U	< 1	< 1	< 1	< 1	< 1	< 1	-	-
**Essential**	Ca	6145	6802	5474	7166	11126	7343	2211	30
	Co	5.7	6.6	5.0	5.8	7.7	6.2	1.0	17
	Cu	< 1	< 1	< 1	< 1	1	< 1	-	-
	Cr total	1.0	1.0	1.0	1.0	2.0	1.2	0.45	37
	Fe	1625	1829	1439	1611	2149	1731	271.7	16
	Mg	687	799	589	721	1045	768	172	22
	Mn	3.5	3.9	3.2	3.7	5.2	3.9	0.77	20
	Ps	85.6	86.4	83.4	75.9	112	88.7	13.7	15
	K	1033	1127	914	1100	1526	1140	230.9	20
	Si	1504	3010	3183	2767	2537	2600	659.7	25
	S	599	552	614	543	548	571	32.8	5.7
	Zn	2.5	2.8	2.1	2.5	4.0	2.8	0.73	26

Among the toxic elements, in all samples, Cd, Pb, As, Ce, La, U, Th and Sb resulted < LOQ. Te and Rb concentrations were 21.7±1.84 and 2.2 ±0.45 **μ**g/g; Al and Ti concentrations were 929±186 and 5267±403.3 **μ**g/g respectively. The mean concentration of Ti was significantly higher than that of other toxic metals; the concentration trend was Ti>>Al>>Te>>Sr>Ba>Rb>Sn>>Ni>Th>Hg, and the concentrations range from 0.0502 (Hg) to 5267 **μ**g/g (Ti).

Among the non-toxic elements, only Cu was consistently < LOQ. All the other determined elements were consistently > LOQ; P, S and Si concentrations were 88.7±13.7, 571±32.8 and 2600±659.7 **μ**g/g, respectively; Mg and Ca concentrations were 768±172 and 7343±2211 **μ**g/g respectively; K and Fe were 1140±230.9 and 1731±271.7 **μ**g/g respectively. The mean concentration of Ca was significantly higher than that of other non-toxic elements; the concentration trend was Ca>>Si>Fe>K>>Mg>S>>P>>Co>Mn>Zn>Cr>Cu and the concentrations ranged from <1 (Cu) to 7343 **μ**g/g (Ca).

The element concentrations found in this study were consistent with those reported by other authors [12–14]. The Cd concentrations found in the sample were consistent with those reported by Wu et al. [34] in cigarettes of the same brands. Indeed, Cd concentrations ranged from 1 to 1.6 **μ**g/g (mean: 1.28±0.17 **μ**g/g) in cigarettes [34] and <1 **μ**g/g in butts (this paper). In Pelit et al. [35] Cd, Cu, Zn and Mn concentrations ranged from <0.75 to 5.80, 9.8 to 102, 10.7 to 125 and 21.2 to 233 mg/kg, respectively in Turkish tobacco leaves and from <0.03 to 1.65, 1.47 to 7.45, 3.93 to 23.9 and 10.1 to 104 **μ**g/g, respectively in the butts. In the present study, it was observed that metal concentrations in the butts were generally lower than those in cigarettes; thus, it was concluded that the metals, although present in the butts, are not completely retained in this part of the cigarette.

### Leached elements

The elements that were considered for leachability evaluation were those with concentrations > LOQ in the original sample of cigarette butts as reported in Table 2 (21 of 30 elements: Al, Ba, Ca, Co, Cr, Fe, Hg, K, Mg, Mn, Ni, P, Rb, S, Si, Sn, Sr, Te, Ti, Tl, Zn).

Tables 3 and 4 show the extraction (%) (mean of three replicates) of every element in three fractions (1, 2 and 3) of five different leachants in the batch extraction and column percolation test respectively. The extraction percentage was obtained by the ratio between the mean content of every element extracted by a leachant and its mean content in the sample prepared for the leaching test. The extraction percentage of Co, Hg, Te, Ti and Tl were not calculated because the concentrations of these elements, although > LOQ in the butts, were < LOQ in all leachates.

**Table 3:** Element % extraction (mean of three analyses) in three fractions (1, 2 and 3) of five different leachants in the batch extraction test (A = 0,4 M MgCl_2_; B = CH_3_COOH + CH_3_COONa pH = 5; C = CH_3_COOH pH = 2.5; D = 0.02 M HNO_3_ + 30% H_2_O_2_, ratio 3:5; E = NaOH pH = 8).

Element	A1	A2	A3	B1	B2	B3	C1	C2	C3	D1	D2	D3	E1	E2	E3
**Al**	0.156	0.293	0.265	0.004	0.126	0.143	0.878	1.76	1.39	0.279	0.571	0.283	0.115	0.312	0.196
**Ba**	4.82	13.5	21.0	0.099	2.05	2.74	10.1	14.8	8.82	5.15	7.05	4.75	1.43	3.34	4.22
**Ca**	3.66	9.29	7.85	0.099	51.8	38.1	49.5	55.2	26.2	9.65	25.6	18.7	1.66	3.49	2.06
**Cr Total**	3.61	1.20	1.20	0.261	3.59	2.28	2.88	4.79	2.56	0.966	9.66	0.644	0.972	0.000	0.000
**Fe**	0.069	0.167	0.008	0.002	0.053	0.044	0.107	0.161	0.139	0.032	0.090	0.023	0.022	0.047	0.013
**P**	20.9	39.6	26.2	0.567	27.8	25.0	27.2	37.6	21.4	21.8	30.8	14.1	7.59	12.4	9.52
**Mg**	0.470	0.470	0.470	0.013	8.50	6.57	8.98	10.4	5.59	5.88	8.40	3.92	4.55	6.48	0.506
**Mn**	17.5	33.1	25.2	0.474	26.3	20.3	26.2	31.0	15.1	8.22	15.3	9.02	2.89	4.98	2.69
**Ni**	12.4	65.0	2.99	0.559	3.02	12.5	5.01	7.18	0.548	0.552	7.29	0.552	0.555	2.50	0.555
**K**	28.4	45.6	31.9	0.771	26.9	19.1	25.8	29.5	13.9	24.0	27.8	10.7	15.8	30.8	15.4
**Si**	0.130	0.322	0.203	0.003	0.003	0.003	0.327	0.417	0.273	0.201	0.294	0.213	0.012	0.106	0.097
**Sn**	1.11	1.39	6.66	0.030	1.50	2.11	26.2	7.37	2.95	6.54	2.97	33.9	12.0	12.0	5.68
**Sr**	4.95	12.4	7.82	0.134	19.9	14.6	22.2	25.2	11.3	7.18	14.7	9.41	3.69	6.63	4.59
**Zn**	16.0	13.6	32.5	0.401	55.4	36.3	76.4	60.7	54.5	3.89	16.0	23.5	17.3	16.1	20.0
**S**	6.38	11.1	7.77	0.173	14.7	13.4	17.4	19.7	9.93	10.6	16.2	8.12	4.70	8.03	4.56
**Rb**	11.3	16.1	11.3	0.307	12.1	8.71	11.7	13.9	6.80	10.7	13.3	6.15	7.95	15.0	7.25

**Table 4:** Element % extraction (mean of three analyses) in three fractions (1, 2 and 3) of five different leachants in the percolation column test (A = MgCl_2_ 0,4 M; B = CH_3_COOH + CH_3_COONa pH = 5; C = CH_3_COOH pH = 2.5; D = HNO_3_ 0.02 M + 30% H_2_O_2_, ratio 3:5; E = NaOH pH = 8).

Element	A1	A2	A3	B1	B2	B3	C1	C2	C3	D1	D2	D3	E1	E2	E3
**Al**	0.413	0.378	0.338	0.167	0.044	0.079	1.24	2.65	2.14	1.72	1.08	0.70	0.54	0.52	0.23
**Ba**	12.6	12.6	6.70	8.98	4.13	<0.101	24.7	16.5	7.82	15.0	7.93	5.45	6.69	3.29	2.21
**Ca**	9.03	5.00	2.91	56.35	56.4	26.5	100	56.6	9.13	21.1	18.1	16.0	2.86	2.51	0.09
**Cr Total**	1.98	1.98	0.992	1.65	0.661	3.64	4.69	5.02	4.69	1.94	3.23	<0.65	2.60	0.32	2.27
**Fe**	0.106	0.106	0.058	0.070	0.046	0.099	0.238	0.367	0.245	0.28	0.14	0.04	0.06	0.12	0.02
**P**	1.48	3.49	1.52	7.38	6.13	2.46	11.2	6.16	2.448	6.03	5.55	1.70	1.71	2.06	0.31
**Mg**	0.517	0.672	0.517	14.8	7.64	0.517	18.7	10.6	0.523	13.4	0.71	1.51	9.94	5.12	0.51
**Mn**	8.04	8.04	3.05	27.7	25.4	13.7	44.3	29.3	7.52	16.6	12.2	30.9	9.69	5.09	3.49
**Ni**	12.8	12.8	3.97	38.7	23.3	14.8	23.3	21.7	8.53	33.4	21.7	25.5	43.1	20.6	4.67
**K**	33.2	14.6	5.33	62.7	22.0	1.88	67.3	27.2	4.37	55.4	19.6	1.43	58.5	36.2	2.36
**Si**	0.061	0.011	0.008	<0.003	<0.003	<0.003	0.056	0.036	0.015	0.03	0.03	0.01	<0.003	<0.003	<0.003
**Sn**	7.63	7.63	10.7	7.33	2.75	8.54	11.1	7.11	2.47	3.88	1.23	7.75	3.89	8.39	4.79
**Sr**	2.39	2.39	0.102	26.0	21.3	7.48	45.3	26.3	3.76	15.2	11.0	6.56	9.34	4.59	1.80
**Zn**	129	129	74.1	146	92.0	118	153	114	62.9	75.7	50.9	19.1	113	52.1	36.8
**S**	1.60	1.11	0.347	3.26	2.36	0.973	4.78	2.53	0.704	3.46	1.90	0.68	2.39	1.64	0.27
**Rb**	7.58	7.58	1.08	28.7	12.4	1.44	30.9	13.1	3.11	25.9	9.77	1.42	22.7	12.6	2.23

### Statistical analysis

Descriptive statistics, as mean, standard deviation and percentage, are reported for each element measured; when quantification is < of LOQ (censored data), a half values of LOQ has been considered. Multiple analysis of variance (MANOVA) was used to assess association among extraction (batch or column), leaching solutions (5 different solutions) and fractions (1, 2, 3), considered as predictive factors and elements measured. MANOVA considered only principal effect; post -hoc analysis were performed using LSD test. IBM SPSS (Ver. 20) software for Windows was used for data analysis, and significance was set at p<0.05.

## Discussion

By results obtained, can be observed that in the batch extraction test, for 12 of the elements that were analyzed, fraction 2 (cumulative ratio, L/S = 6.7 ml/g) extracted, for all leachants, a greater amount of the element than fractions 1 and 3, but the post-hoc statistical analysis shows that this statement is significant (p <0.05) only for K and Rb, while fraction 2 is significantly richer only than 1 for P.

On the contrary, in the column percolation test, for 14 of the elements, fraction 1 (ratio L/S = 3.3) extracted, for all leachants, a greater amount of the element than fractions 2 and 3, but this is statistically significant (p <0.05) only for Mg, K, Sr, Rb, while for Ba, P, Sr, Zn the fraction 1 is significantly more effective (p <0.05) than only 3. It can therefore be stated that 8 (50%) of the elements analyzed are mainly extracted from the first two eluate fractions.

Table 5 shows the element extraction as the total % leached (cumulative release % of three fractions) in five different leachants in the batch extraction and column percolation test and the ratio (c/b) between the % leachate in the column percolation test (c) and the % leachate in the batch extraction test (b) for every leachant. In the last column of Table 5, the mean ratio (c/b) was shown for every element. The ratio between the total % extraction in the column percolation test and the total % extraction in the batch test was > 1 for all the elements, except for Ca, P, S and Si. Based on these data, it seems that the amount of leachate is higher in the column percolation test than in the batch extraction test. Indeed, a significant difference exists in the extraction efficiency of the two systems (F_21; 2_ = 71491; p <0.001), but post-hoc analysis show significantly a greater efficacy of percolation in the column (p <0.05) only for the elements Al, Fe, Ni and Zn, while batch solubilization is better (p <0.05) for P, Si, and S.

**Table 5:** Element % extraction (sum of fractions 1, 2 and 3) in five different leachants in the batch extraction and column percolation test and ratio (c/b) between the % leachate in the column percolation test (c) and that found in the batch extraction test (b).

	0.4 M	MgCl_2_	pH = 6.5	CH_3_COOH	+CH_3_COONa	pH = 5	CH_3_COOH	pH = 2.5		0.02 M	HNO_3_ +	H_2_O_2_	NaOH	pH = 8		Mean Ratio
Element	Batch	Column	c/b	Batch	Column	c/b	Batch	Column	c/b	Batch	Column	c/b	Batch	Column	c/b	c/b
**Al**	0.715	1.13	1.58	0.273	0.29	1.06	4.04	6.02	1.49	1.13	3.51	3.11	0.62	1.29	2.07	1.86
**Ba**	39.3	31.8	0.810	4.89	13.2	2.70	33.8	49.0	1.45	16.9	28.4	1.68	9.00	12.2	1.36	1.60
**Ca**	20.8	16.9	0.801	90.0	92.4	1.03	98.3	96.9	0.99	53.9	55.2	1.02	7.21	5.47	0.76	0.92
**Cr Total**	6.01	4.96	0.830	6.13	5.95	0.97	10.2	14.4	1.41	11.3	5.81	0.51	0.97	5.19	5.34	1.81
**Fe**	0.244	0.27	1.11	0.099	0.21	2.12	0.406	0.85	2.09	0.14	0.47	3.36	0.08	0.19	2.32	2.20
**P**	86.7	6.49	0.070	53.3	16.0	0.30	86.1	19.9	0.23	66.7	13.3	0.20	29.6	4.08	0.14	0.19
**Mg**	1.41	1.70	1.21	15.1	22.9	1.52	25.0	29.8	1.19	18.2	15.6	0.86	11.5	15.6	1.36	1.23
**Mn**	75.8	19.1	0.250	47.0	66.8	1.42	72.4	81.1	1.12	32.5	59.7	1.84	10.6	18.3	1.73	1.27
**Ni**	80.3	29.6	0.37	16.0	76.9	4.79	12.7	241	19.0	8.39	110	13.1	3.61	68.3	18.9	11.2
**K**	100	53.2	0.530	46.8	86.6	1.85	69.2	98.9	1.43	62.5	76.4	1.22	61.9	97.1	1.57	1.32
**Si**	0.655	0.08	0.120	0.01	0.010	1.00	1.02	0.11	0.11	0.71	0.070	0.10	0.22	0.01	0.05	0.25
**Sn**	9.16	25.9	2.83	3.64	18.6	5.11	36.6	20.7	0.57	43.4	12.9	0.30	29.6	17.1	0.58	1.88
**Sr**	25.2	4.88	0.190	34.6	54.7	1.58	58.6	75.4	1.29	31.3	32.7	1.04	14.9	15.7	1.05	1.03
**Zn**	62.0	333	5.37	92.2	357	3.87	97.5	331.5	3.40	43.4	146	3.36	53.4	202	3.78	3.96
**S**	25.3	3.06	0.120	28.2	6.60	0.23	47.1	8.02	0.17	35.0	6.04	0.17	17.3	4.29	0.25	0.19
**Rb**	38.7	16.2	0.420	21.1	42.6	2.02	32.4	47.1	1.45	30.2	37.1	1.23	30.2	37.5	1.24	1.27

**Fig 1:**
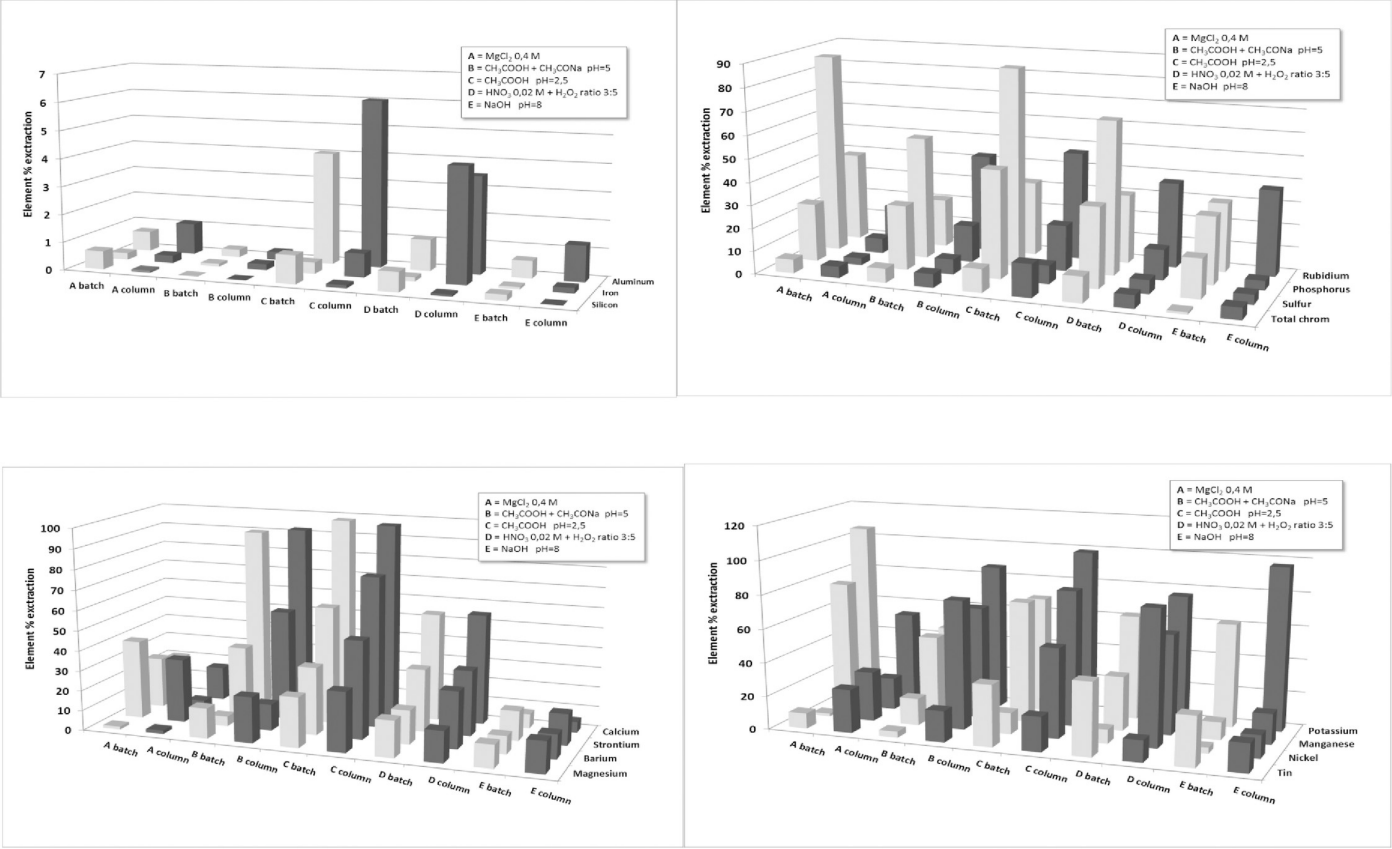
Comparison between the total % extraction of every leachant (A = 0.4M MgCl_2_ pH 6.5; B = CH_3_COONa pH 5; C = CH_3_COOH pH 2.5; D = 0.02M HNO_3_ and 30% H_2_O_2_ (ratio 3:5) pH <1; E = NaOH pH 8) in batch extraction test and that obtained in column percolation test.

Fig 1 shows a comparison between the total % extraction in the batch test and that obtained in the column percolation test for all the elements reported in Table 5 for each leachant.

A significant difference (F_84; 20_ = 3.99; p <0.001) was also found on the extraction efficiency of the 5 solvents in the two systems. In the batch extraction test, leachant C (CH_3_COOH, pH 2.5 as organic acids found in soil) carried out achieved the max % extraction for 7 of the elements that were analyzed. Statistical analysis confirm (p <0.05) the assumption above reported only for 5 elements (Al, Ca, Fe, Sr and Zn) on the total of the determinate elements.

In the column percolation test, leachant C (CH_3_COOH, pH 2.5) showed the max % extraction for 13 of elements, but statistically significant (p <0.05) reaches only for 10 elements (Al, Ba, Ca, Cr, Fe, P, Mn, Ni, Si, Sr) on the total of the determinate elements.

Overall, leachant C seems to have a greater capacity to dissolve the elements than the other leachants; which may be due to the acidic environment and the complexing capacity of acetic acid.

Weakly acidic solutions (leachant A-pH 6.5 and leachant B-pH 5.0 to simulate acid rainwater), or alkaline environments (leachant E-pH 8 to simulate a leachate coming from a landfill) do not promote element leachability.

As illustrated in Table 5 and in Fig 1, the findings of this study suggest that differences in pH within the typical range of precipitation (pH 6.5–4) have no appreciable effect on the metal concentrations leached from cigarette butts.

### Limitation

However as a limit of this work can be noted that in several cases, our experiments showed low accuracy (ex: Zn extraction > 100%) and low repeatability due the complexity of the tested material and the leaching tests; however, they still can provide valuable information on the potential release of toxic elements from cigarette butts. Moreover, when trace elements (heavy metals) were examined, our tests did not yield any results, mainly due to the very low concentrations of these elements in leachates. Hence, other more sensitive techniques should be used to measure toxic trace elements.

## Conclusions

Understanding what happens to the metals present in cigarette butts when they are dispersed in the environment is important because it allows us to assess their effects on the local biota and the environment in general. In this work, the results of two different leaching tests, the batch extraction and column percolation tests, were compared. To reproduce a worst-case scenario and not to underestimate the leachability of toxic elements processes of acidification and oxidation were simulated using synthetic solutions instead of demineralized water.

The results seem to show that the acid environment and the complexing capacity of the acetic acid have a greater capacity to dissolve the elements than other leachants. Indeed, weakly acidic solutions or alkaline environments promote lesser element leachability. Furthermore, the column percolation test, which maybe provides the most frequent condition of the leaching process (percolation in a static position) that occurs under real conditions, is better for Al, Fe, Ni and Zn. While the batch extraction, which simulates the tumbling and trampling to which butts can be subjected once dispersed in the environment, is more effective for P, Si and S.

In conclusion, the results show that discarded cigarette butts are point sources for prolonged metal contamination and the rapid release of multiple metals from these butts increases the potential for acute harm to local organisms.

## Supporting information

S1 Dataset (XLS)
